# Germination-Arrest *Bacillus subtilis* Spores as An Oral Delivery Vehicle of Grass Carp Reovirus (GCRV) Vp7 Antigen Augment Protective Immunity in Grass Carp (*Ctenopharyngodon idella*)

**DOI:** 10.3390/genes11111351

**Published:** 2020-11-14

**Authors:** Rui Sun, Miao Zhang, Hui Chen, Yao Wei, Degang Ning

**Affiliations:** 1 Huai’an Research Center, Institute of Hydrobiology, Chinese Academy of Sciences, Huai’an 223005, China; sunr8904@163.com (R.S.); zm775727189@163.com (M.Z.); 18352862463@163.com (Y.W.); 2 China Jiangsu Center of Aquatic Animal Disease Prevention and Control, Nanjing 210036, China; chenhuijsbf@163.com; 3 CAS Key Laboratory of Algal Biology, Institute of Hydrobiology, Chinese Academy of Sciences, Wuhan 430072, China

**Keywords:** *Bacillus subtilis*, germination-arrest spore, surface display, GCRV, oral vaccination, protective immunity, grass carp

## Abstract

Oral vaccination is a practical method for the active immunization of farmed fish in the matter of animal welfare and handling costs. However, it always shows insufficient protective immunity, mainly due to antigen degradation in the gastrointestinal tract (GIT). *Bacillus subtilis* spores have been shown to be able to protect surface-display heterologous antigens against degradation. Neverthless, the spores can germinate in GIT, which causes loss of the antigens with spore coat disassembly. Here, we developed a novel surface display system using the *B. subtilis* spore coat proteins CotB and CotC as anchors for the heterogenous antigen, and the germination-controlling genes *cwlJ* and *sleB* as the ectopic integration sites for the fusion genes. Using this display system, we engineered germination-arrest spores displaying the model antigen Vp7 of grass carp reovirus (GCRV) on their surface. Oral vaccination of the engineered spores could confer immune protection against GCRV in grass carp (*Ctenopharyngodon idella*) via eliciting adaptive humoral and cellular immune responses. Most importantly, the germination-arrest spores were shown to significantly augment immunogenicity and protection above the engineered spores based on the existing surface display system. Therefore, the presently reported antigen expression strategy opens new and promising avenues for developing oral vaccines for the immunization of farmed fish species.

## 1. Introduction

Aquaculture has been the fastest-growing culture field for years, and its yield is still rising. As a consequence, increasing culture densities cause severe stress, which conversely makes fish more susceptible to infections. Recently, frequent outbreaks of infectious diseases give rise to severe economic losses for the aquaculture industry worldwide [1]. Vaccination is considered to be an effective means to prevent and control infectious diseases in fish populations [2]. To date, vaccines available for some aquaculture fish species are given via intraperitoneal injection, immersion, or oral route [3]. Injection vaccination is most effective, but impractical nor achievable, due to high labor intensity and sever stress [4]. Oral vaccination would be an ideal method for vaccine delivery in the matter of animal welfare and handling costs, while it always shows insufficient protection mainly due to antigen degradation in GIT [5]. Thus, an ideal delivery system of specific antigens is crucial for developing effective and feasible oral vaccines of fish [6].

A desired antigen delivery system for oral vaccines must include safety, protection against antigen breakdown, and denaturation [3]. Probiotic bacterial strains are considered to be very promising tools to meet these needs [7]. The gram-positive soil bacterium *Bacillus subtilis* is a non-pathogen, and its spore form is currently used as a probiotic for both human and animal consumption [8]. More importantly, the *B. subtilis* spores offer unique resistance properties and can survive extreme environments [9]. Due to good a safety record and high resistance to harsh environmental conditions, the *B. subtilis* spores are considered as a potential vehicle for oral vaccination [9,10]. Based on the well-documented structure and assembly of *B. subtilis* spores, a spore surface display technique has been developed using a spore coat protein as an anchor partner for specific antigens and a nonessential gene as an integrative site for fusion genes in the chromosome [10]. Using genetic modification, a chimeric gene consisting of a fusion between a *B. subtilis* anchor gene and an open reading frame (ORF) encoding a protective antigen is first constructed. Subsequently, a recombinant strain can be engineered via the introduction of the chimera into the *B. subtilis* chromosome. When the spores form, the chimeric protein is displayed on their surface. It has been demonstrated that the spore-displayed antigens could transit across GIT in mammals and induce immune responses [11,12,13,14]. However, oral administration of the recombinant spores usually confers limited protective immunity. One main reason is considered that spores have been shown to germinate in GIT, causing loss of the surface-displayed antigens with spore coat disassembly [15,16].

Spore germination is triggered by the induction of nutrient and non-nutrient molecules, as well as by some specific physical treatment [17]. Nutrient germinants include L-alanine and a mixture of L-asparagine, fructose, glucose, and KCl (AFGK), and the best-known non-nutrient germinant is dipicolinic acid (DPA) in 1:1 chelate of Ca^2+^ (DPA-Ca) [17,18]. The two types of germinants act quite differently for the germination of *B. subtilis* spores. The germination induced by nutrient germinants requires specific spore receptors that are encoded by the genes of the ger-family operons [17,19]; the germinant-receptor interaction then induces a series of germination events, including rehydration of the spore’s dehydrated cytoplasm, excretion of endogenous spore DPA, and cortex hydrolysis. However, the germinant DPA-Ca does not require the receptor to induce spore germination. In *B. subtilis*, two function-redundant cortex-lytic enzymes, named SleB and CwlJ, are naïve to be responsible for cortex hydrolysis, and to be required for spore germination induced by nutrient or non-nutrient germinants [17,20,21].

To make *B. subtilis* spores more efficiently deliver specific antigens in the GIT environment, we devised a strategy by which germination-arrested spores were engineered to be used as a delivery vehicle of an oral vaccine of fish. For this, we first developed a novel spore surface display system that consists of a pair of integrative vectors using spore coat proteins CotB and CotC as anchors, and the germination-controlling genes *cwlJ* and *sleB* as the integrative sites. To evaluate the feasibility of this strategy, we engineered germination-arrested spores surface displaying the model protective antigen Vp7 of grass carp reovirus (GCRV) [22], one of the most serious pathogenic aquareovirus which can cause lethal hemorrhagic disease in grass carp (*Ctenopharyngodon idella*) [23]. The engineered spores were demonstrated to able to significantly augment immunogenicity and protection in fish orally vaccinated.

## 2. Materials and Methods

### 2.1. Strains and Growth Conditions

Plasmid amplifications for subcloning experiments were performed with *Escherichia coli* strain DH5α or BL21(DE3) (Invitrogen, Carlsbad, CA, USA). *B. subtilis* 168 (*trp^-^*) (*Bacillus* Genetic Stock Center,) was used for the preparation of recombinant spores by spore surface display systems. The *E. coli* and *B. subtilis* strains were routinely cultivated in Luria-Bertani (LB) broth (37 °C). When necessary, the cultures of *E. coli* were supplemented with ampicillin (Ap, 50 µg/mL) or kanamycin (Km, 50 µg/mL). The derivatives of *B. subtilis* 168 (*trp^-^*) were grown in the medium with erythromycin (Em, 0.4 µg/mL) or kanamycin (Km, 10 µg/mL).

### 2.2. Construction of Plasmids and Recombinant B. Subtilis Strains

The plasmids listed in Table 1 were made using standard molecular biology techniques [24]. The primers used for constructing plasmids were synthesized by Sangon Biotech (Shanghai, China) and listed in Table S1. All cloned sequences were confirmed by DNA sequencing.

**Figure 1:**
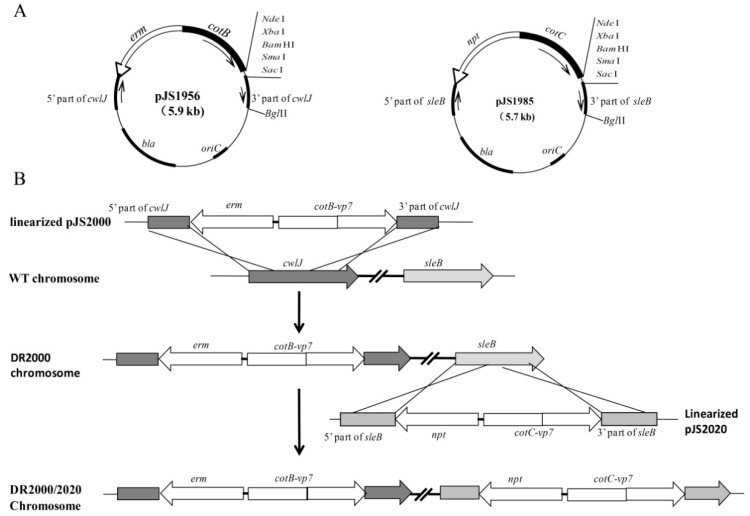
Schematic diagram showing the construction of the recombinant *B. subtilis* strains. (**A**) The genetic structure of the integrative platform vectors pJS1956 and pJS1985. (**B**) The flow chart for the construction of the strain DR2000/2020.

We first constructed integrative platform plasmids pJS1956 and pJS1985 (Figure 1A). The *cotB* gene, containing its natural promoter (*P_cotB_*) and the 825-bp DNA fragment encoding the first 275 amino acids, was amplified from *B. subtilis* chromosome DNA using the primers cotB-1 and cotB-2. The *erm* gene conferring erythromycin resistance (Em^r^) was amplified from the plasmid pMutin 2 [26] using the primers *erm*-1 and *erm*-2. The fusion PCR method [27] was employed for developing the recombinant fragment *erm-cotB* using the primer pair of *erm*-2 and cotB-2. The resulting fragment *erm-cotB* was subcloned into pMD18-T (TaKaRa, Dalian, China), producing the plasmid pJS1692. A fragment containing the *cwlJ* gene was amplified from *B. subtilis* chromosome DNA using the primers cwlJ-1 and cwlJ-2, and cloned between the *Pvu*II sites of pUC18 producing pJS1951. The plasmid pJS1951 was linearized by reverse PCR using the primers cwlJ-R1 and cwlJ-R2. The resulting fragment was digested using *Pst*I and *Sac*I, and linked to the *erm-cotB* fragment excised from pJS1692 by the same enzymes, yielding pJS1956 (containing the recombinant fragment *cwlJ::erm-cotB*). For the integrative platform vector pJS1985 (Figure 1A), the *cotC* gene, including the *cotC* promoter (*P_cotC_*) and the 516-bp coding sequence without stop codon, was amplified with the primers cotC-1 and cotC-2. The *npt* gene conferring kanamycin resistance (Km^r^) was amplified from the plasmid pUB110 [28] using the primers npt-1 and npt-2. The recombinant fragment *npt-cotC* was made by the fusion PCR method [27] using the primers npt-2 and cotC-2, and cloned to pMD18-T generating pJS1691. The *sleB*-containing fragment was amplified using the primer sleB-1 and sleB-2, and cloned between the *Pvu*II sites of pUC18 producing pJS1976. The linearized pJS1976 was obtained by the reverse PCR method using the primers sleB-R1 and sleB-R2, and digested with *Pst*I and *Sac*I. The resulting fragment was linked to the *npt-cotC* fragment from pJS1691, generating pJS1985 (containing the recombinant fragment *sleB::npt-cotC)*.

According to the sequence of the gene *vp7* (GenBank No. AF403396), encoding the antigen Vp7 of the reference strain 873 of type II GCRV, an 840-bp ORF of the *vp7* gene was codon-optimized for the expression in *B. subtilis* and synthesized by Sangon Biotech (Shanghai, China). The synthetic fragment was digested with *Nde*I and *Sac*I, and subcloned into the *Nde*I/*Sac*I sites of pJS1985 and pJS1956, yielding pJS2000 (containing the recombinant fragment *cwlJ::erm-cotB-vp7*) and pJS2020 (containing the recombinant fragment *sleB::npt-cotC-vp7*), respectively. In addition, the *Nde*I/*Sac*I-digested *vp7* was cloned into pJS700 [25], an integrative platform vector used in the existing display system, obtaining pJS1947 (containing the recombinant fragment *amyE::erm-cotB-vp7*). For over-expression of Vp7 tagged with six histidine residues at its N-terminus (His_6_-Vp7), the fragment *vp7* was cloned into pET28a (Novegen, Carlsbad, CA, USA) between *Nde*I and *Sac*I sites yielding pJS1621.

To construct recombinant *B. subtilis* strains, the integrative plasmids (Table 1) were separately linearized by digestion with *Bgl*II and then introduced into *B. subtilis* strain 168 (*trp^-^*) by transformation following the previously described procedures [29]. The transformants with correct integration were verified by genomic PCR using corresponding primers.

### 2.3. Spore Preparation and Germination Analysis

Spores were prepared from cultures using the Difco sporulation medium (DSM), concentrated by centrifugation, and treated with lysozyme to destroy any residual vegetative cells, as previously described in detail [25]. Then the spores were purified by a wash with water, and treatment at 65 °C for 45 min to kill residual vegetative cells or germinated spores [30]. The number of the purified spores was measured by direct count with a Burker chamber under a microscope (Leica DFC300FX with 40 × lense), and adjusted to a density of more than 1 × 10^1^^1^ spores/mL. Aliquots were frozen at known concentrations at −20 °C till use. Colony formation assays were used to assess spore germination. One hundred microliters of the spore suspension with a density of about 4 × 10^8^ spores/mL was spread on LB medium agar plates following appropriate dilution as required. The plates were incubated at 37 °C for 24 h, and colonies were counted.

### 2.4. Preparation of the Recombinant Vp7 Protein and Rabbit Anti-Vp7 Antibody

The recombinant His_6_-Vp7 protein was expressed from pJS1621 in *E. coli* BL21 (DE3) by induction with IPTG, and purified by nickel affinity column chromatography using Ni-NTA His•Bind^®^ Resins (Novagen, Carlsbad, CA, USA). The eluted protein was checked for integrity by 15% SDS-PAGE, and its concentration was determined using Protein Assay kit (Bio-Rad, Hercules, CA, USA). The purified His_6_-Vp7 protein was used to raise the anti-Vp7 antibody in a New Zealand rabbit via an intraperitoneal route with 10 μg His_6_-Vp7 in sterile 0.85% NaCl formulated with 50% (*v/v*) Freund complete adjuvant (Sigma-Aldrich, St. Louis, MO, USA) in a total volume of 200 μL per dose. After 30 days of primary immunization, the animals were boosted at 10-day intervals for 1–2 times. One week after the last immunization, sera were collected and checked for antibody specificity at a dilution of 1:2000 by Western blot analysis.

### 2.5. Immunoblotting Analysis of Spore Coat Proteins

Spore coat proteins were extracted from the purified spores using an SDS-DTT extraction buffer, as described in detail elsewhere [30]. For western blot analysis, 50 µg of the extracted proteins were fractionated on 10% SDS-PAGE, electro-transferred to polyvinylidene difluoride (PVDF) membrane (Pall, Hercules, CA, USA) using minitransfer blot (Bio-Rad, Hercules, CA, USA). The Western blot assays were performed using previously described protocols [25]. Reactive bands were detected with enhanced chemiluminescence (ECL) reagent (Sigma-Aldrich, St.Louis, Missouri, USA) and exposed to X-film as previously described [25]. For dot blot analysis, 2 µL of 10-fold dilution of the coat proteins with a defined amount was spotted on the FVDF membrane. After treatment, as described in Western blot analysis, the filter was stained with Metal Enhanced DAB Substrate kit (Solarbio, Beijing, China). Images were analyzed densitometrically using Image J.

### 2.6. Immunofluorescence Analysis

Five hundred microliters of spore suspension at a density of 1 × 10^8^ spores/mL were used for immunofluorescence analysis. The spores were blocked for 15 min with 2% (*w/v*) bovine serum albumin (BSA) in PBS (pH7.4) at room temperature, and then washed three times with PBS. The washed spores were resuspended in 500 µL of PBS, and incubated with rabbit anti-Vp7 antibodies (1: 1000) for 45 min at room temperature. After further washed, the spores were incubated with goat anti-rabbit IgG-Cy5 conjugates (1: 1500, Sangon, Shanghai, China) for 45 min at room temperature. After an additional wash, the labeled spores were resuspended in 500 μL of PBS, and immediately analyzed with a fluorescence microscope and fluorospectrophotometer. For immunofluorescence microscope analysis, 10 μL of the spore suspension was dropped on a microscope slip, covered with a coverslip, and then viewed under Leica DFC300FX fluorescence microscope (Leica, Somls, Germany) using a Sapphire filter set (Exciter D620–40, dichroic 660DCLP, and emitter D670/40) (Chroma Technology, Bellows Falls, VT, USA). Images were captured using a Leica DFC300FX high sensitivity digital camera assisted by the software Leica QWin Lite S/W (Leica, Somls, Germany). For fluorospectrophotometer analysis, the fluorescence emission spectra of the immunolabelling spores were measured using a Cary Eclipse Fluorescence Spectrophotometer (Varian, Salt Lake, UT, USA). The excitation wavelength was set at 649 nm, and the emission wavelength was from 600 to 800 nm.

### 2.7. Preparation of Experimental Fish and Spore-Coated Feed Pellets

Healthy grass carps (50 ± 5 g mean weight) were kindly obtained from Jiangsu Tianshen Co., Ltd. (Nanjing, China). After acclimated to the laboratory conditions for two weeks, fish were randomly distributed into 50 L tanks (*n* = 30). The temperature of water in each tank was maintained at 25–28 °C and sufficient oxygen were supplied with the oxygen-increasing system. The fish were fed with feed pellets (Tianshen, China) at amounts equivalent to 2% of fish body weight every day. All experimental procedures that involved animal manipulation were approved by the Animal Research and Ethics Committee of the Institute of Hydrobiology of the Chinese Academy of Sciences (No. HDB20170403). For the preparation of spore-coated feed pellets, the purified spores were mixed with the pellets (Tianshen, China) in the light of 1.0×10^10^ spores/g feed, and incubated on ice for 15 min to allow absorption of the spore suspension. The pellets were coated with plant oil, and stored at 4 °C until further use.

### 2.8. Oral Administration, Sample Collection, and Challenge

For oral administration of *B. subtilis* spores, fish in the groups were fed with the spore-coated pellets at amounts equivalent to 2% of fish body weight (equivalent to 1.0 × 10^10^ spores/fish). After 7 days of primary immunization, the vaccinated fish were boosted following the same dose as the primary oral vaccination. On day 0, 7, 14, 21, and 28 after the boost immunization, blood and head kidney were collected from 5 fish in each group. For this, the fish randomly selected were anesthetized via immersion in the water added with clove oil, then the sample collection was performed according to described elsewhere [31].

The GCRV strain HA741, a virulent strain of GCRV-II, was kindly provided by Dr. Rui Yuan of Jiangsu center of aquatic animal disease prevention and control, and used for challenge tests. The virus was cultured in *Ctenopharyngodon idella* kidney (CIK) cell. The CIK cell culture methods and 50% tissue culture infective doses (TCID50) of the virus were performed according to the established protocols [32]. Challenge experiments for fish orally vaccinated with the recombiant spores were conducted by intraperitoneal injection with a dose of 100TCID_50_ GCRV on day 14 after the boost immunization. Also, the positive and negative (naïve) control groups were included, which had been fed with uncoated pellets followed by intraperitoneal injection with 100TCID_50_ GCRV and with PBS (pH 7.4), respectively. The mortality and clinical signs of the challenged fish were recorded daily for two weeks postchallenge. All experiments were performed for three replicates. The relative percent survival (RPS) was calculated by the following formula: RPS = {1 − (% mortality rate (treatment group)/% mortality rate (positive control)) × 100.

### 2.9. Indirect Enzyme-Linked Immunosorbent Assay (ELISA) of Anti-Vp7 IgM Antibody

The anti-Vp7 IgM levels in the collected serum samples were measured by indirect ELISA, as described elsewhere [33]. Briefly, the purified His_6_-Vp7 was used as an antigen to coat the microplate overnight at 4 °C. Blocking was performed by incubating the plate for 2 h with a blocking buffer (5% skimmed milk in PBS). Subsequently, 100 µL of the serum samples that had been serially diluted 10-fold with PBS were added and were incubated for 1 h at 37 °C. The plates were washed with PBS for 3 times, and incubated with 100 µL of rabbit anti-IgM antibodies (1: 2000 dilution in PBS containing 5% skimmed milk, Zoonbio Biotech, Nanjing, China) for 1 h at 37 °C. After washed, 100 µL of HRP-conjugated goat anti-rabbit IgG (1: 5000 dilution in PBS containing 5% skimmed milk, Zoonbio Biotech, Nanjing, China) were added, and incubated for 1 h at 37 °C. Following additional wash steps, color development was performed using tetramethylbenzidine (TMB, Bios, Beijing, China) for 10–15 min at room temperature after washing. Finally, 2 M H_2_SO_4_ was used to stop the reaction, and the absorbance of each well at 450 nm was read with a precision microplate reader (Molecular Devices, San Jose, CA, USA). The anti-Vp7 IgM levels in sera were expressed as absorbance (OD_450_).

### 2.10. Total RNA Isolation, cDNA Synthesis and Quantitative Reverse Transcription PCR (qRT-PCR)

Total RNA was extracted from the head kidney samples using Trizol reagent (TaKaRa, Dalian, China) following the manufacturer’s protocols. The cDNA was then synthesized using Reverse transcriptase kit (TaKaRa, Dalian, China), and was stored at –80 °C for further use. To quantitative determination of gene expression, qRT-PCR analysis was performed using SYBR Premix Ex Taq II kit (TaKaRa, Dalian, China). The determined genes included two cellular immune-related genes, cluster of differentiation 4-like (CD4L) and major histocompatibility complex class II (MHC-II), and two inflammation-related genes, interleukin-1β (IL-1β) and tumor necrosis factor-α (TNF-α). Their expression levels were normalized using the housekeeping gene β-actin [34]. Specific primers were designed and listed in Table S1. The amplifications were carried out in a 12.5 μL reaction volume with CFX96 Real-Time PCR Detection System (Bio-Rad, St. Louis, MO, USA). To quantify qRT-PCR data, the 2–ΔΔCt method modified by Rao et al. [35] was used. The errors for the ΔΔCt were obtained by least square error propagation of the standard deviation for the individual qRT-PCR measurements performed in triplicates.

### 2.11. Statistical Analysis

The data were analyzed by one-way ANOVA after normalization (SPSS 16.0 software USA). Differences in antibody titers and transcription levels of the immune-related genes were analyzed with Duncan’s test. The data of mortality rate and relative percentage survival were transformed to square-root arcsine values before performing the differences test with SPSS statistical software. In all analyses, *p* < 0.05 were considered significant.

## 3. Results

### 3.1. Recombinant B. Subtilis Strains

To construct recombinant *B. subtilis* strains that can produce germination-arrested spores expressing heterologous antigens of interest on their surface, we developed a novel display system. First, we constructed a pair of integrative platform vectors pJS1956 and pJS1985 as described in Materials and Methods (Figure 1A). Both vectors harbor a ColE1 origin, a multi-clone site (MCS) at the end of the *cotB/cotC* gene, and a β-lactamase selection marker *bla* in *E. coli*. In pJS1956 *cotB* was used as the anchor gene, encoding a 31.1-kDa CotB fusion partner, *erm* as the selection marker in *B. subtilis*, and *cwlJ* as the integration fragment; however, in pJS1985 *cotC* was used as the anchor gene, encoding a 14.6-kDa CotC partner, *npt* as the selection marker in *B. subtilis*, and *sleB* as the integration fragment. These two integrative platform vectors could be used to generate the translational fusion of the anchor partner to genes of interest by cloning them into the MCS. Here, we constructed the recombinant plasmids pJS2000 and pJS2020 using the *vp7* gene of GCRV, encoding the outer capsid protein Vp7. The Vp7 protein (29.8 kDa) is a putative protective antigen against GCRV infection in grass carp [22].

Using the pairs of the integrative plasmids, the recombinant *B. subtilis* strains were made by progressively transforming the strain 168 (*trp^-^*). The ectopic integration of the chimeric genes accompanied by the gene Em^r^ or Km^r^ from the integrative plasmids caused the simultaneous inactivation of *cwlJ* and *sleB* in the resulting strains. Taking the strain DR2000/2020 as an example (Figure 1B), the linearized pJS2000 was introduced into the *B. subtilis* 168 cells, and Em^r^ transformants that arose from double crossover events between the plasmid and the bacterial chromosome were identified by genomic PCR for the ectopic integration of the recombinant at the *cwlJ* locus (Figure S1A). The correct clone was then transformed with the linearized pJS2020 with selection for kanamycin, and the Km^r^ transformant with desired integration at the *sleB* locus was termed the strain DR2000/2020 (Table 1, Figure S1A). Similarly, the recombinant strains DR1956/1985 and DR2000/1985 were made with two plasmid pairs of pJS1956/pJS1985, and pJS1956/pJS2020, respectively. In these recombinant strains, both genes *cwlJ and sleB* are inactivated simultaneously by the ectopic integration of the recombinant segments *Em^r^-cotB-vp7* and *Km^r^-cotC-vp7* in DR2000/2020, *Em^r^-cotB-vp7* and *Km^r^–cotC* in DR1956/2020, and *Em^r^-cotB* and *Km^r^ –cotC* in DR1956/1985 (Table 1).

In addition, we used the existing display system [25] for the construction of the recombinant strain DR1947 with the plasmid pJS1947 (Table 1). In the strain DR1947, the Em^r^-*cotB-vp7* fragment was introduced in the chromosome at the *amyE* locus, causing the inactivation of *amyE* (Figure S1B,C). The strain DR700 was also constructed using the integrative plate vector pJS700 [25], in which the Em^r^-*cotB* segment was integrated at the *amyE* locus (Table 1).

### 3.2. Spore Germination of Recombinant B. Subtilis Strains

To determine the germination of the spores from the recombinant strains, we tested their ability to germinate on LB plates. The spores of DR1956/1985, DR2000/1985, and DR2000/2020 were almost not able to germinate, but those of DR700 and DR1947 could (Figure S2). These suggested that the recombinant spores made by the novel spore display system were strongly impaired in germination due to the inactivation of *cwlJ* and *sleB*. To quantitatively determine the germination of the recombinant spores, we chose the spores of DR1956/1985 and DR700 as the representatives made by the novel display system and the existent display system, respectively. The germination rate of the DR1956/1985 spores (0.0044%) was significantly lower than that of the WT spores (95%) or the DR700 spores (104%) (Table 2). This was consistent with the previous study where the mutant spores lacking CwlJ and SleB exhibited 0.001% of the germination rate of wild-type spores [20]. Thus, the recombinant spores based on the novel display system show a germination-arrested phenotype, while those made by the existing display system have a cogenic wild-type phenotype.

### 3.3. Expression and Localization of Vp7 in the Recombinant Spores

**Figure 2:**
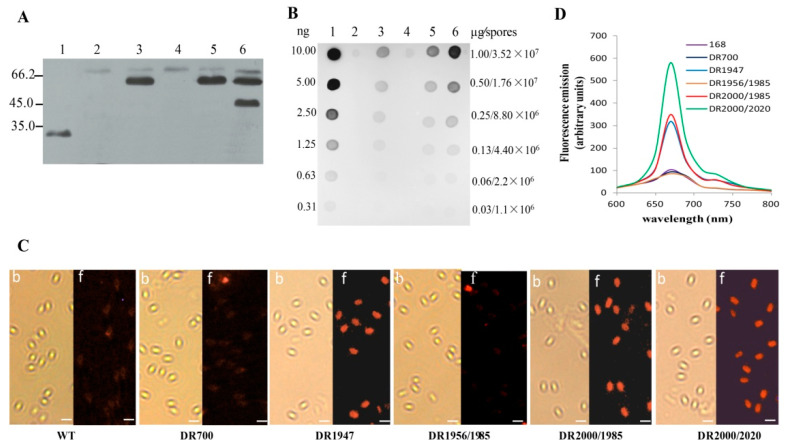
Detection of the Vp7 antigen in the recombinant *B. subtilis* spores. (**A**) Western blot analysis of the Vp7 antigen in the spore coat proteins. Lane 1, the purified His_6_-Vp28; lanes 2–6, the coat proteins extracted from the spores of the recombinant strains DR700, DR1947, DR1956/1985, DR2000/1985, and DR2000/2020, respectively. Molecular weight markers (kDa) are indicated at the right. (**B**) Dot blot analysis for the amount of the Vp7 antigen in the recombinant spore coat. The amounts of His_6_-Vp7 (lane 1) and the coat proteins from the spores of DR700 (lane 2), DR1947 (lane 3), DR1956/1985 (lane 4), DR2000/1985 (lane 5), and DR2000/2020 (lane 6) were indicated. (**C**) Immunofluorescent detection of the Vp7 antigen on the spore surface. The immunofluorescence-labeled spores were visualized under bright field (b) and fluorescence (f) microscopy. Scale bars indicate 1 μm. (**D**) Quantitative analysis of the Cy5 fluorescence on the spore surface with fluorospectrophotometer.

To determine whether the Vp7 antigen was expressed in the spore coat, we performed Western blot analysis using anti-Vp7 antibodies via reacting with the fusion protein CotB-Vp7 or CotC-Vp7. As shown in Figure 2A, no band was visualized in the coat proteins extracted from the spores of DR700 and DR1956/1985 (lanes 2 and 4); however, a protein with a molecular weight of ~60 kDa were shown in the spore coat of the strains DR1947 and DR2000/1985 (lanes 3 and 5), and two proteins with molecular masses of ~60 kDa and ~44 kDa were observed in the spore coat of the strain DR2000/2020 (lane 6). According to the calculated molecular masses of CotB-Vp7 (60.7 kDa) and CotC-Vp7 (44.4 kDa), the results suggested the DR2000/2020 spores simultaneously expressed both chimera CotB-Vp7 and CotC-Vp7 in their coat, the spores of DR2000/1985 and DR1947 expressed only CotB-Vp7, while the spores of DR700 and DR1956/1985 did not express the Vp7 antigen.

To quantitatively determine the Vp7 antigen in the coat of the recombinant spores, we performed dot blot experiments using anti-Vp7 antibodies and the defined amounts of the coat proteins. The results were shown in Figure 2B. Based on a densitometric analysis, and the molecular mass of His_6_-Vp7 (31.8 kDa), the amount of Vp7 in the spore coat was estimated (Figure 2B). Since an average of 2.84 × 10^−8^ μg of total coat proteins was reproducibly extracted from each spore under our experimental conditions, we calculated that each spore of DR1947 and DR2000/1985 expressed approximately 1.78 × 10^3^ molecules (corresponding to 8.78 × 10^−11^ μg) and 1.96 × 10^3^ molecules (equivalent to 9.66 × 10^−11^ μg) of Vp7, respectively. In the case of the DR2000/2020 spores, each spore had about 3.21 × 10^3^ molecules (equivalent to 15.38 × 10^−11^ μg) of Vp7, about 1.8 times as much as those in each spore of DR1947 or DR2000/1985. These suggested that the use of double fusion partners (CotB and CotC) in the novel display system increased the abundance of the Vp7 antigen expressed in the recombinant spores.

To localize the Vp7 antigen, we used immunofluorescence microscopy to observe the spores incubated with anti-Vp7 primary antibodies, followed by Cy5-conjugated secondary antibodies. As observed under bright field microscopy, all recombinant strains were able to produce mature spores (Figure 2C). The spores of DR1947, DR2000/1985, and DR2000/2020 showed clear red fluorescence under the fluorescence field, while those of WT, DR700, and DR1956/1985 did not (Figure 2C). These suggested that the germination-arrested spores, similar to the congenic wild-type spores, could display Vp7 on their surface. In addition, we measured the fluorescence intensity of the Cy5-labeled spores using fluorospectrophotometer (Figure 2D). The spores of DR1947, DR2000/1985, and DR2000/2020, exhibited a remarkable Cy5-specific fluorescence peak at 670 nm (Figure 2D). The fluorescence value of the DR2000/2020 spores (551 ± 37) was about 1.6-fold higher than that of the spores of DR1947 (317 ± 15) or DR2000/1985 (338 ± 17), while there was no significant difference between the spores of DR1947 and DR2000/1985. These data are consistent with those from the dot blot analyses, further demonstrating that the recombinant spores based on the novel display system could increase the abundance of the Vp7 antigen on their surface.

### 3.4. Immune Responses of Grass Carp Following Oral Immunization

**Figure 3:**
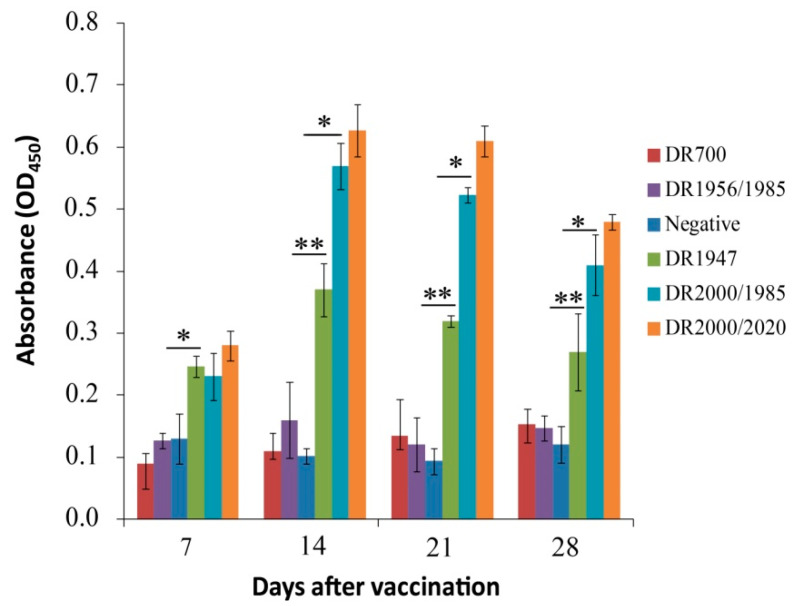
ELISA analysis of the anti-Vp7 IgM titers in the sera of grass carp. Serum samples were collected on the indicated days post oral vaccination. The results represent the means and standard deviations from three independent experiments. The comparison between the DR1947 group with the Naive group or the DR1956/2020 group was shown. Values that were significantly different from the control were indicated by asterisks (* *p* < 0.05, ** *p* < 0.01).

It has been demonstrated that oral administration of the spores with a specific protective antigen is able to confer humoral and cellular immune responses in mammals [11,12,13,14]. Because the IgM antibody in fish functions as a major humoral immunity factor [36,37,38], we first determined the anti-Vp7 IgM titer in sera from fish fed with the spore-coated pellets. The results were shown in Figure 3. The IgM levels in fish given with the spores not expressing Vp7 (DR700 and DR1956/1985), similar to those in the negative group, were shown no change during the whole test course. In fish orally vaccinated with the Vp7-expressing spores, the IgM titers were significantly increased on day 7 after oral vaccination, and reached peaks on day 14, then dropped from day 21 to 28. Nevertheless, after 7 days (on days 14, 21, and 28) of oral vaccination, the increased levels of anti-Vp7 IgM in fish given with the spores of DR2000/1985 were significantly higher than those with the DR1947 spores (*p <* 0.05), but slightly less than those with the DR2000/2020 spores (*p >* 0.05). These results indicated that oral administration of the Vp7-expressing spores could elicit anti-Vp7 IgM immune response in grass carp, while the germination-arrested spores expressing Vp7 on their surface (DR2000/1985 and DR2000/2020) could confer more efficient humoral immunity than the congenic wild-type spores surface displaying Vp7 (DR1947). In addition, we observed that this humoral immunity depended on the dose of the Vp7 antigen orally administrated according to the results of dot blot analysis (Figure 2B,D).

**Figure 4:**
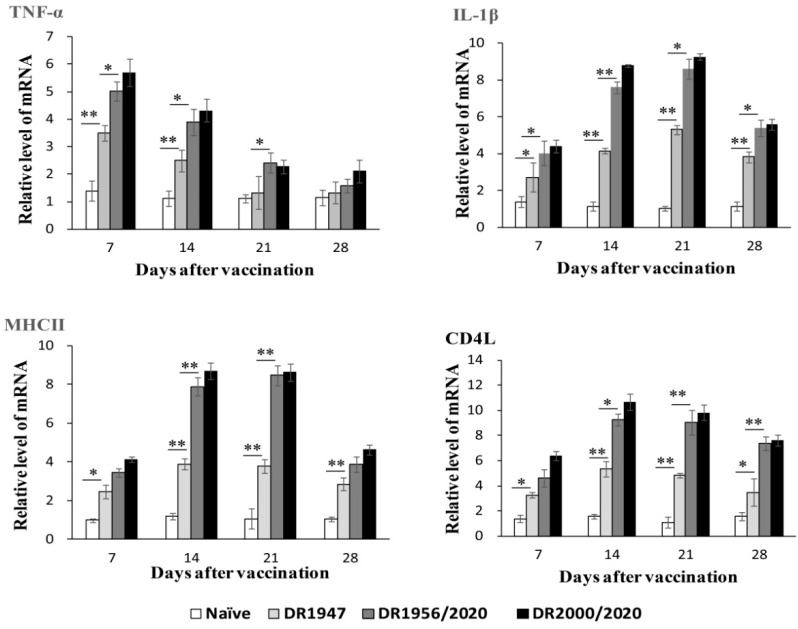
qRT-PCR analysis of the transcriptional response of immune-related genes in the head kidney of grass carp. Data are means for three independent assays and presented as the means ± SD. The comparison between the group DR1947 with the Naive group or the group DR1956/2020 was shown. Values that were significantly different from the control were indicated by asterisks (* *p* < 0.05, ** *p* < 0.01).

In fish, both CD4L and MHC II are critical molecular markers of adaptive cellular immunity, while IL-1β and TNF-α are important inflammatory factors involved in regulating immune functions [39,40]. To determine the cellular immune response, we used qRT-PCR analysis to examine the expression levels of the immune factors in the head kidney, an important immune organism in fish. As shown in Figure 4, the transcription of the genes was significantly upregulated in the head kidney from fish in the groups orally vaccinated with the spores of DR1947, DR2000/1985, and DR2000DR2020 relative to that in the naïve group, as well as in the groups orally dosed with the spores of DR700 and DR1956/1985. Similar to the IgM response, the transcriptional upregulation observed in the group DR2000/1985 was somewhat lower than that in the group DR2000DR2020, but significantly above that in the group DR1947. In addition, the transcriptional upregulation between the genes showed different temporal profiles. The upregulation peak of TNF-α occurred on day 7 after oral vaccination, but IL-1β, MHCII, and CD4L did on day 14 (Figure 4). These results suggested that oral vaccination of the germination-arrested spores expressing Vp7 could trigger higher levels of cellular immunity and inflammatory response than that of the congenic wild-type spores expressing Vp7.

### 3.5. Protection of Grass Carp against GCRV after Oral Administration

To test the biological function of the immune responses, we determined the protection of grass carp against GCRV by challenge experiments. According to the temporal profiles of the immune responses, we chose to perform the challenge tests on day 14 after oral vaccination. As a negative control, fish had 100% survival rate within 14 days (Figure S3). In the positive group, GCRV challenge caused fish with a cumulative mortality rate of about 74.45% (Table 3, Figure S3). In the groups DR1947, DR2000/1985, and DR2000/2020, the cumulative mortality rates of fish were 53.33%, 36.67%, and 32.22%, and the corresponding RPSs were 28.09%, 50.66%, and 56.73%, respectively (Table 3). In comparison, the mortality rate of the group DR1947 was significantly higher than that of both groups DR2000/1985 and DR2000/2020 (*p* < 0.01), while the group DR2000/1985 was slightly higher than the group DR2000/2020 (Table 3). These suggested that the Vp7-expressing spores given by the oral route are therefore protective, and the germination-arrested spores can deliver the Vp7 antigen more efficiently than the congenic wild-type spores.

## 4. Discussion

Several attributes, including safety, extreme robustness, and its use as a probiotic, make the *B. subtilis* spores a particularly promising delivery vehicle of an oral vaccine. The *Bacillus subtilis* spores displaying heterogenous antigens on their surface have been shown to induce immune protection in mammals through oral administration [11,12,13,14]. However, such immunity is considered to be adversely affected by spore germination, known to occur inside the GIT. In addition, the *B. subtilis* spore so far has been rarely reported as a delivery vehicle of an oral vaccine in fish. In this study, we engineered the germination-inhibited spores which display the immunogen Vp7 of GCRV on their surface, and used them to assess the effect of spore germination on immune protection in grass carp by oral vaccination.

To prepare the Vp7-expressing germination-inhibited spores, we developed a novel spore display system. Using this system, we successfully obtained the *B. subtilis* strains, which were able to produce germination-arrested spores expressing the Vp7 antigen on their surface (DR2000/1985 and DR2000/2020, Figure 2). Comparing to the existing spore display system, the novel display system: (1) Can be used to produce the germination-inhibited spores expressing the specific antigen; (2) is able to enhance the abundance of the heterogenous antigen expressed on the spore surface due to the simultaneous use of CotB and CotC as the anchore proteins; (3) allows each spore to display two heterogenous antigens on its surface, and to be used for developing bivalent oral vaccines. Indeed, the effect of spore germination on the immune response has been studied using the *B. subtilis* strain lacking *gerD* to produce germination-deficient spores expressing a specific antigen in mice, and the same study concluded that spore germination was not related to the immune response to the surface-displayed specific antigen [41]. The gene *gerD,* encoding a putative lipoprotein, was originally considered to be responsible for spore germination at an early stage in response to various known germinants [42]. However, a later study confirmed that the *gerD*-deleted spores only respond slower than the wild-type spores, and they can ultimately germinate, outgrow, and form colonies [43]. Here we showed that the spores made by the novel display system were strongly impaired in germination due to the simultaneous inactivation of *cwlJ* and *sleB*. Thus, the use of these spores as a delivery vehicle of oral antigen allows us to better investigate the effect of spore germination on immune efficacy.

Using the germination-inhibited spores expressing the Vp7 antigen, we investigated the effect of spore germination on immune response and protection against GCRV in grass carp by oral vaccination. We found that an oral dose of the germination-inhibited spores could confer more efficient immune protection than that of the congenic wild-type spores. This conclusion first comes from the evidence on the increase of the anti-Vp7 IgM levels and the upregulation of MHC II and CD4L in the fish (Figure 4). In teleost fish, IgM is the most abundant immunoglobulin in blood and in mucosal-associated lymphoid tissue, and is considered to be the most important immunoglobulin in systemic immunity for disease resistance [36,37,38]. MHC II plays a vital role in the adaptive immune response by presenting antigenic peptides to CD4^+^ T lymphocytes [44], and CD4L is critical molecular markers for defining the T helper cell subset [45], which provides the basis for addressing the involvement of T cells [46]. Furthermore, the enhanced immune response is also supported by a quantitative assay of expression of IL-1β and TNF-α in fish orally administrated with the Vp7-expressing germination-inhibited spores. Both IL-1β and TNF-α are important inflammatory factors in cells (e.g., macrophages/monocytes), which mediates the inflammatory responses and regulates immune functions [39,40]. Consistent with the immunological response, the immune protection against GCRV in the fish dosed with the Vp7-expressing germination-inhibited spores are significantly higher than that induced by oral vaccination of the Vp7-expressing wild-type spores (Table 3). These suggest that the germination-inhibited spores as the oral delivery vehicle of the antigen Vp7, could augment immune protection against GCRV via eliciting higher levels of adaptive humoral and cellular immunity. However, whether specific mucosal immunity in the fish gut is involved in this immune protection remain to be evaluated. In teleost fish, the immunoglobulin isotype, IgZ or IgT has been demonstrated to be ubiquitous in the intestine, skin, and gill and to play an important role in mucosal immunity [47,48]. Therefore, the IgZ or IgT response to the Vp7 antigen orally delivered by the recombinant spores deserves further investigation in our future studies.

In this study, we also observed that the immune protection induced by oral vaccination of the recombinant spores depends on the dose of the Vp7 antigen. Although an identical amount (2 × 10^11^ spores) of the recombinant spores was given, the spores expressing both chimera CotB-Vp7 and CotC-Vp7 (corresponding to 30.88 μg of the Vp7 antigen) conferred more efficiently immune protection than those expressing only the chimeras CotB-Vp7 (corresponding to about 19.32 μg of the Vp7 antigen). Consistently, in an early study, the immune protection against GCRV in grass carp immunized with *E. coli*-expressed Vp7 via either injection or bath rout, were shown to be enhanced with the increase of the Vp7 dose [49]. These imply that there is plenty of room for improvement of immune protection by further investigating oral dose, as well as the cycle, frequency, and route of booster immunization.

## 5. Conclusions

In conclusion, the germination-arrested spores are a more efficient delivery vehicle of oral vaccine than the wild-type spores. The immune protection effects achieved with the presently reported antigen expression strategy open new and promising avenues for developing oral vaccines for the passive immunization of farmed fish species. In addition, since the *B. subtilis* spores also are considered as a good vector to express and immobilized enzymes of interest on their surface, the novel display system has the potential to be used as a platform to develop immobilized enzymes.

## Figures and Tables

**Table 1:** Plasmids and strains used in this paper.

Plasmid	Characteristic Property or Genotype		Origen or Reference
pJS700	An integrative vector containing the integrative fragment *amyE::erm-cotB*		[25]
pJS1947	A derivative of pJS700, containing the integrative fragment *amyE::erm-cotB-vp7*		This study
pJS1956	An integrative vector containing the integrative fragment *cwlJ::erm-cotB*		This study
pJS1985	An integrative vector containing the integrative fragment *sleB::npt-cotC*		This study
pJS2000	A derivative of pJS2016, containing the integrative fragment *cwlJ::erm-cotB-vp7*		This study
pJS2020	A derivative of pJS1976, containing the integrative fragment *sleB::npt-cotC-vp7*		This study
pJS1621	The vp7 gene in pET28a		This study
***B. subtilis***	**Relevant Genotype**	**Expressed Fusion Antigen ^1^ on Spore Surface**	**Origen or Reference**
168 (*trp^-^*)	Wild type (WT)	-	BGSC ^2^
DR700	*amyE::erm-cotB*	-	This study
DR1947	*amyE:erm-cotB-vp7*	CotB-Vp7, 60.7 kDa	This study
DR1956/1985	*cwlJ::erm-cotB; SleB::npt-cotC*	-	This study
DR2000/1985	*cwlJ::erm-cotB-vp7; sleB::npt-cotC*	CotB-Vp7, 60.7 kDa	This study
DR2000/2020	*cwlJ::erm-cotB-vp7; sleB::npt-cotC-vp7*	CotB-Vp7, 60.7 kDa; CotC-Vp7, 44.4 kDa	This study

^1^ Molecular weight of protein chimera was estimated according to its amino acid sequence and determined by SDS-PAGE analysis. ^2^ *Bacillus* Genetic Stock Center.

**Table 2:** Germination efficiency of the *B. subtilis* spores on LB plate ^1.^

Spore	No. of Spores ^2^	CFU	Germination Efficiency ^3^
WT168	4.64 × 10^8^	4.58 × 10^8^	99%
DR700	4.32 × 10^8^	4.51 × 10^8^	104%
DR1956DR1985	4.85 × 10^8^	2.14 × 10^4^	0.0044%

^1^ Data reported here were the average of three independent experiments.^2^ The number of spores was counted under a microscope.^3^ Defined as the ratio of the CFU numbers on LB agar plates and initially counted the number of the purified spores.

**Table 3:** Cumulative mortality rate and RPS of grass carp challenged with GCRV ^1.^

Group	Cumulative Mortality Rate (%)	RPS(%)	*p* Value ^2^
Positive	74.45 ± 3.14	--	--
DR1947	53.33 ± 2.72	28.09 ± 1.79	0.036/--
DR2000/1985	36.67 ± 1.57	50.66 ± 0.30	0.008/0.031
DR2000/2020	32.22 ± 3.14	56.73 ± 2.82	0.007/0.024

^1^ Values represent the means and standard deviations from three independent experiments. ^2^ *p* values were obtained by comparing the cumulative mortality rate between the groups DR1947, DR2000/1985, and DR2000/2020 with the positive group or the group DR1947 with the groups DR2000/1985 and DR2000/2020, respectively.

## Supplementary Materials

The following are available online at https://www.mdpi.com/2073-4425/11/11/1351/s1, Figure S1: Identification of the recombinant *B. subtilis* strains by PCR or amylase analysis, Figure S2: Spore germination of the recombinant strains and the wild-type strain 168 on LB plates, Figure S3: Cumulative survival rate of grass carp after GCRV challenge, Table S1: The primers used in this study.

## References

[1] Brudeseth B.E., Wiulsrod R., Fredriksen B.N., Lindmo K., Lokling K.E., Bordevik M., Steine N., Klevan A., Gravningen K. (2013). Status and future perspectives of vaccines for industrialised fin-fish farming. Fish Shellfish Immunol..

[2] Dhar A.K., Manna S.K., Thomas Allnutt F.C. (2014). Viral vaccines for farmed finfish. Virusdisease.

[3] Hoelzer K., Bielke L., Blake D.P., Cox E., Cutting S.M., Devriendt B., Erlacher-Vindel E., Goossens E., Karaca K., Lemiere S. (2018). Vaccines as alternatives to antibiotics for food producing animals. Part 2: New approaches and potential solutions. Vet. Res..

[4] Mutoloki S., Munang’andu H.M., Evensen O. (2015). Oral vaccination of fish—Antigen preparations, uptake, and immune induction. Front. Immunol..

[5] Munang’andu H.M., Mutoloki S., Evensen O. (2015). An overview of challenges limiting the design of protective mucosal vaccines for finfish. Front. Immunol..

[6] Embregts C.W., Forlenza M. (2016). Oral vaccination of fish: Lessons from humans and veterinary species. Dev. Comp. Immunol..

[7] Unnikrishnan M., Rappuoli R., Serruto D. (2012). Recombinant bacterial vaccines. Curr. Opin. Immunol..

[8] Hong H.A., Duc L.H., Cutting S.M. (2015). The use of bacterial spore formers as probiotics. FEMS Microbiol. Rev..

[9] Ferreira L.C., Ferreira R.C., Schumann W. (2005). *Bacillus subtilis* as a tool for vaccine development: From antigen factories to delivery vectors. An. Acad. Bras. Cienc..

[10] Oggioni M.R., Ciabattini A., Cuppone A.M., Pozzi G. (2003). *Bacillus* spores for vaccine delivery. Vaccine.

[11] Lee J.S., Poo H., Han D.P., Hong S.P., Kim K., Cho M.W., Kim E., Sung M.H., Kim C.J. (2006). Mucosal immunization with surface-dislayed severe acute respiratory syndrome coronavirus spike protein on *Lactobacillus casei* induces neutralizing antibodies in mice. J. Virol..

[12] Barnes A.G., Cerovic V., Hobson P.S., Klavinskis L.S. (2007). *Bacillus subtilis* spores: A novel microparticle adjuvant which can instruct a balanced Th1 and Th2 immune response to specific antigen. Eur. J. Immunol..

[13] Knecht L.D., Pasini P., Daunert S. (2011). Bacterial spores as platforms for bioanalytical and biomedical applications. Anal. Bioanal. Chem..

[14] Tavares Batista M., Souza R.D., Paccez J.D., Luiz W.B., Ferreira E.L., Cavalcante R.C., Ferreira R.C., Ferreira L.C. (2014). Gut adhesive *Bacillus subtilis* spores as a platform for mucosal delivery of antigens. Infect. Immun..

[15] Casula G., Cutting S.M. (2002). *Bacillus* probiotics: Spore germination in the gastrointestinal tract. Appl. Environ. Microbiol..

[16] Bernardeau M., Lehtinen M.J., Forssten S.D., Nurminen P. (2017). Importance of the gastrointestinal life cycle of *Bacillus* for probiotic functionality. J. Food Sci. Technol..

[17] Setlow P. (2014). Germination of spores of *Bacillus* species: What we know and do not know. J. Bacteriol..

[18] Olguin-Araneda V., Banawas S., Sarker M.R., Paredes-Sabja D. (2015). Recent advances in germination of *Clostridium* spores. Res. Microbiol..

[19] Atluri S., Ragkousi K., Cortezzo D.E., Setlow P. (2006). Cooperativity between different nutrient receptors in germination of spores of *Bacillus subtilis* and reduction of this cooperativity by alterations in the GerB receptor. J. Bacteriol..

[20] Paidhungat M., Ragkousi K., Setlow P. (2001). Genetic requirements for induction of germination of spores of *Bacillus subtilis* by Ca^2+^-dipicolinate. J. Bacteriol..

[21] Setlow P. (2013). Summer meeting 201—When the sleepers wake: The germination of spores of *Bacillus* species. J. Appl. Microbiol..

[22] Shao L., Sun X., Fang Q. (2011). Antibodies against outer-capsid proteins of grass carp reovirus expressed in *E. coli* are capable of neutralizing viral infectivity. Virol. J..

[23] Rangel A.A., Rockemann D.D., Hetrick F.M., Samal S.K. (1999). Identification of grass carp haemorrhage virus as a new genogroup of aquareovirus. J. Gen. Virol..

[24] Sambrook J., Fritsch E.F., Maniatis T. (1989). Molecular Cloning: A Laboratory Manual.

[25] Ning D., Leng X., Li Q., Xu W. (2011). Surface-displayed VP28 on *Bacillus subtilis* spores induce protection against white spot syndrome virus in crayfish by oral administration. J. Appl. Microbiol..

[26] Vagner V., Dervyn E., Ehrlich S.D. (1998). A vector for systematic gene inactivation in *Bacillus subtilis*. Microbiology.

[27] Wang H., Postier B.L., Burnap R.L. (2002). Optimization of fusion PCR for in vitro construction of gene knockout fragments. Biotechniques.

[28] McKenzie T., Hoshino T., Tanaka T., Sueoka N. (1987). A revision of the nucleotide sequence and functional map of pUB110. Plasmid.

[29] Cutting S.M., Horn P.B.V., Harwood C.R., Cutting S.M. (1990). Genetic analysis. Molecular Biological Methods for Bacillus.

[30] Nicholson W.L., Setlow P., Harwood C.R., Cutting S.M. (1990). Sporulation, germination and outgrowth. Molecular Biological Methods for Bacillus.

[31] Wang N., Wu Y., Pang M., Liu J., Lu C., Liu Y. (2015). Protective efficacy of recombinant hemolysin co-regulated protein (Hcp) of *Aeromonas hydrophila* in common carp (*Cyprinus carpio*). Fish Shellfish Immunol..

[32] Fang Q., Seng E., Ding Q., Zhang L. (2008). Characterization of infectious particles of grass carp reovirus by treatment with proteases. Arch. Virol..

[33] Zhu B., Liu G., Gong Y., Ling F., Wang G. (2015). Protective immunity of grass carp immunized with DNA vaccine encoding the vp7 gene of grass carp reovirus using carbon nanotubes as a carrier molecule. Fish Shellfish Immunol..

[34] Su J., Zhang R., Dong J., Yang C. (2011). Evaluation of internal control genes for qRT-PCR normalization in tissues and cell culture for antiviral studies of grass carp (*Ctenopharyngodon idella*). Fish Shellfish Immunol..

[35] Rao X., Huang X., Zhou Z., Lin X. (2013). An improvement of the 2^∧^(-delta delta CT) method for quantitative real-time polymerase chain reaction data analysis. Biostat. Bioinforma. Biomath..

[36] Salinas I., Zhang Y.A., Sunyer J.O. (2011). Mucosal immunoglobulins and B cells of teleost fish. Dev. Comp. Immunol..

[37] Bengten E., Wilson M. (2015). Antibody Repertoires in fish. Results Probl. Cell Differ..

[38] Magadan S., Sunyer O.J., Boudinot P. (2015). Unique features of fish immune repertoires: Particularities of adaptive immunity within the largest group of vertebrates. Results Probl. Cell Differ..

[39] Tracey K.J., Cerami A. (1993). Tumor necrosis factor, other cytokines and disease. Annu. Rev. Cell Biol..

[40] Baud V., Karin M. (2001). Signal transduction by tumor necrosis factor and its relatives. Trends Cell Biol..

[41] Mauriello E.M., Cangiano G., Maurano F., Saggese V., De Felice M., Rossi M., Ricca Z. (2007). Germination-independent induction of cellular immune response by *Bacillus subtilis* spores displaying the C fragment of the tetanus toxin. Vaccine.

[42] Pelczar P.L., Igarashi T., Setlow B., Setlow P. (2007). Role of GerD in germination of *Bacillus subtilis* spores. J. Bacteriol..

[43] Moir A., Smith D. (1990). The genetics of bacterial spore germination. Ann. Rev. Microbiol..

[44] Li X., Du H., Liu L., You X., Wu M., Liao Z. (2017). MHC class II α, β and MHC class II-associated invariant chains from Chinese sturgeon (*Acipenser sinensis*) and their response to immune stimulation. Fish Shellfish Immunol..

[45] Somamoto T., Yoshiura Y., Nakanishi T., Ototake M. (2005). Molecular cloning and characterization of two types of CD8alpha from ginbuna crucian carp, *Carassius auratus langsdorfii*. Dev. Comp. Immunol..

[46] Yang M., Wang Y., Wang X., Chen C., Zhou H. (2010). Characterization of grass carp (*Ctenopharyngodon idellus*) Foxp1a/1b/2: Evidence for their involvement in the activation of peripheral blood lymphocyte subpopulations. Fish Shellfish Immunol..

[47] Zhang Y.A., Salinas I., Li J., Parra D., Bjork S., Xu Z., LaPatra S.E., Bartholomew J., Sunyer J.O. (2010). IgT, a primitive immunoglobulin class specialized in mucosal immunity. Nat. Immunol..

[48] Rombout J.H., Yang G., Kiron V. (2014). Adaptive immune responses at mucosal surfaces of teleost fish. Fish Shellfish Immunol..

[49] Hao K., Chen X., Qi X., Zhu B., Wang G., Ling F. (2018). Display of GCRV vp7 protein on the surface of *Escherichia coli* and its immunoprotective effects in grass carp (*Ctenopharyngodon idella*). Fish Shellfish Immunol..

