# Supplementary material for: Germination-Arrest Bacillus subtilis Spores as An Oral Delivery Vehicle of Grass Carp Reovirus (GCRV) Vp7 Antigen Augment Protective Immunity in Grass Carp (Ctenopharyngodon idella)
Source: Genes (Basel). 2020 Nov 14;11(11):1351. doi: 10.3390/genes11111351 (PMC7696455; doi:10.3390/genes11111351)
Supplement: Supplementary file 1 [file genes-11-01351-s001.pdf]

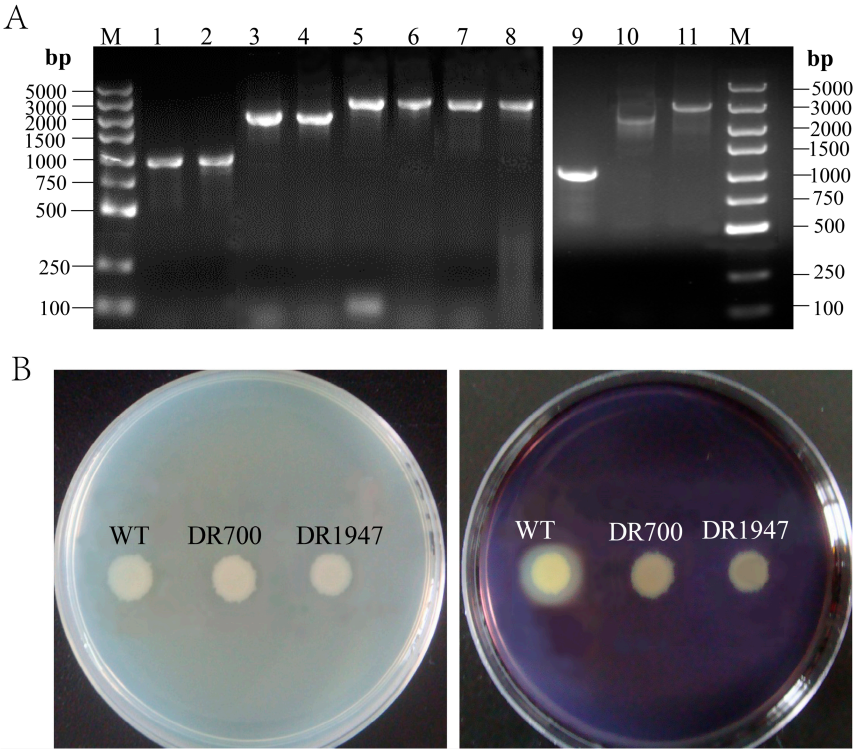

Figure S1 Identification of the recombinant *B.subtilis*. (A) PCR identification of the strains WT, DR1956/1985, DR2000/1985 and DR2000/2020 using the primers sleB-1/sleB-2 (Lanes 1, 3, 5, 7) and the primers cwlJ-1/cwlJ-2(Lanes 2, 4, 6, 8), as well as the strain DR700 and DR1947 using the primer amyE-1/amyE-2 (lanes 10 and 11). (B) Amylase activity analysis of the strain DR700 and DR1947 on the strarch plate stained with iodine solution.

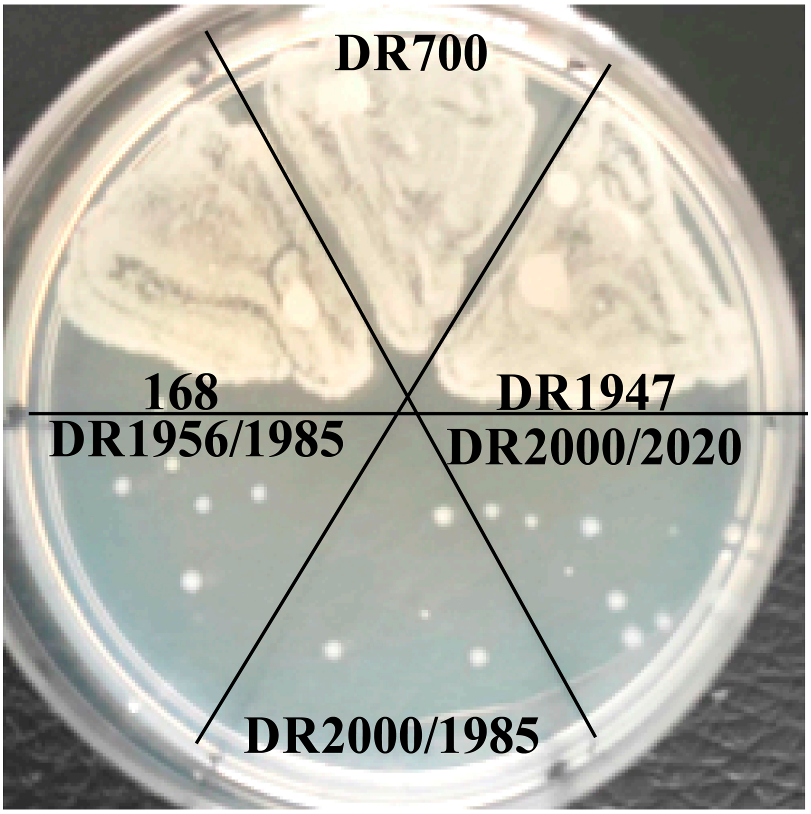

Figure S2 Spore germination of recombinant strains and wild-type strain 168 on LB plates.

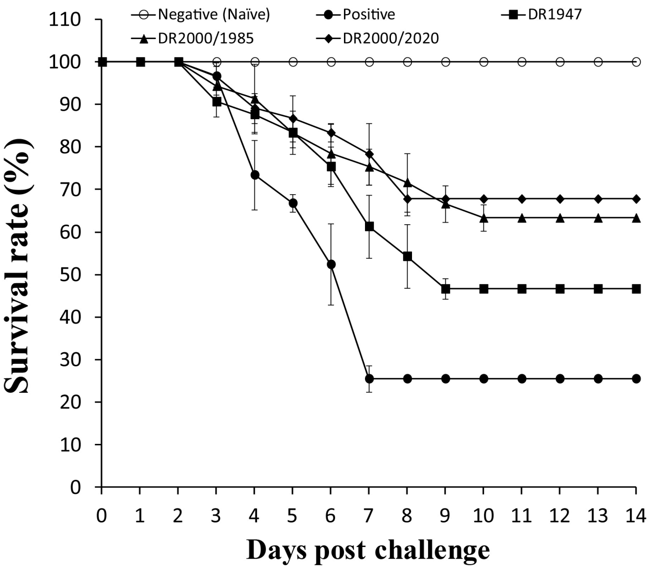

Figure S3 Cumulative survival rate of grass carp after GCRV challenge. Data are means for three assays and presented as the means ± SD (n=30).

Table S1 The primers used in this study

Primer	Sequence (5′–3′)*	Accession number	Restriction site
pUC18-R1	GAGAGAGGAGATCTGCGTAATAGCGAAGAGGCCC		BglII
pUC18-R2	GAGAGAGGAGATCTCGGCGGTTTGCGTATTGGGCG		BglII
cwlJ-1	GAGAGAGGAGATCTCAAAGCCCAGACCAAGCACCAG	BSU02600	BglII
cwlJ-2	GAGAGAGGAGATCTCCCATACATCCAGCAAACCG		BglII
cwlJ-R1	GAGAGAGGACTGCAGTGAGCAATTCGCCCGCAGCC		PstI
cwlJ-R2	GAGAGAGGAGAGCTCGCGAGATAGCGAGCGTGCCC		SacI
sleB-1	GAGAGAGGAGATCTGAGGCTTGAAAAGATGAAGTCC	BSU22930	BglII
sleB-2	GAGAGAGGAGATCTCCTGGTGTTCTTTGTATCCCC		BglII
sleB-R1	GAGAGAGGACTGCAGGCATTCGCTTTAGGCTTCTGCG		PstI
selB-R2	GAGAGAGGAGAGCTCAACAGGATGCGGTAGCAGCG		SacI
cotB-1	AGTTTACATAATATATATTCTAGGAAGTCGGATTAGGC CGTTTGTCCTC	BSU36050	
cotB-2	CATATGGGATGATTGATCATCTGAAGAT		NdeI
cotC-1	CCGGAGTTTACATAATATATATTCTAGGAAGTCCGGTT CAAAATAAATGCATTCCC	BSU17700	
cotC-2	CATATGGTGTTTTTTATGCTTTTTATACTCTACAAC		NdeI
emr-1	GACTTCCTAGAATATATATTATGTAAACTCGAAAAGTG CCACCTGACGTC		
emr-2	AGTTTACATAATATATATTCTAGGAAGTAGCTCCTTGG AAGCTGTCAG		
npt-1	GGACTTCCTAGAATATATATTATGTAAACTCCGTTAAT GCGCCATGACAG		
npt-2	GGAGTTTACATAATATATATTCTAGGAAGTGACGAAAC TGCAAAATATCC		
vp7-1	GGAGAGACATATGCCACTTCACATGATTCCGC		NdeI
vp7-2	GGAGAGAGAGCTCTGATGGTTCCACGTGCAAGTCGAG	AF403396	SacI
IL-11F	GCTGAGGCATCGGAGGCACAT	DQ417927	
IL-11R	TTGGGTCTCGCACCATTTTCTC		
β-actin -1	GATGATGAAATTGCCGCACTG	M25013	
β-actin -2	ACCGACCATGACGCCCTGATGT		
TNF-α-1	TGTGCCGCCGCTGTCTGCTTCACGCT	EU047718	
TNF-α -2	GATGAGGAAAGACACCTGGCTGTAGA		
IL-1β-1	GGAGAATGTGATCGAAGAGCGT	EU047716	
IL-1β-2	GCTGATAAACCATCCGGGA		
MHC II-1	TACTACCAGATTCACTCGG	GU441573	
MHC II-2	CGGGTTCCAGTCAAAGAT		
CD4L-1	ATGTGTCCAGGTGTCATAGT	KX033448	
CD4L-2	GGAATTTTGACTGTATAGGAT		

*Underlined are the recognition sites for the restriction enzymes indicated in the table.
